# Anatomical study of the teres major muscle: description of an additional distal muscle slip

**DOI:** 10.1186/s12891-021-04227-3

**Published:** 2021-04-16

**Authors:** Lukas Ernstbrunner, Malik Jessen, Marco Rohner, Manuel Dreu, Samy Bouaicha, Karl Wieser, Paul Borbas

**Affiliations:** 1 Department of Orthopedics, Balgrist University Hospital, University of Zurich, Forchstrasse 340, 8008 Zurich, Switzerland; 2 Gottfried Schatz Research Center for Cell Signaling, Metabolism and Aging, Macroscopic and Clinical Anatomy, Medical University of Graz, Graz, Austria

**Keywords:** Teres major muscle, Distal teres major slip, Latissimus dorsi muscle, Tendon transfer, Deltopectoral approach

## Abstract

**Background:**

Understanding muscle and tendon anatomy is of tremendous importance to achieve optimal surgical execution and results in tendon transfers around the shoulder. The aim of this study was to introduce and describe an additional distal muscle slip of the teres major (TM).

**Methods:**

Sixteen fresh-frozen cadaver shoulders were dissected with the deltopectoral approach. The ventral latissimus dorsi (LD) tendon was harvested, and the shoulders were analyzed for the presence/absence of a distal teres major slip (dTMs) and its dimensions and relationship with the TM and LD tendons.

**Results:**

The dTMs was identified in 12 shoulders (75%). It was always distal to the TM tendon and visible during the deltopectoral approach. There was a clear separation between the TM proximally and dTMs tendon distally. At the humeral insertion, both tendons had a common epimyseal sheet around the teres major and inserted continuously at the humerus. The mean width of the dTMs tendon at the insertion was 13 ± 4 mm (range, 7–22 mm). The total lengths of the dTMs tendon and LD tendon were 40 ± 7 mm (range, 25–57 mm) and 69 ± 7 mm (range, 57–79 mm), respectively (*p* < 0.001). The dTMs muscle showed direct adhesions in ten shoulders (83%) with the LD muscle.

**Conclusions:**

This is the first macroscopic description of an additional distal slip of the teres major muscle. The dTMs has a separate (distal) but continuous (mediolateral) insertion at the humerus within a common epimyseal sheet around the TM. The dTMs tendon is visible during the deltopectoral approach and can therefore provide a lead structure, particularly in ventral LD transfers with the deltopectoral approach.

## Background

In 1934, L ‘Episcopo first described the teres major (TM) and then the latissimus dorsi (LD) transfer in the treatment of obstetric brachial plexus palsy in six children [1]. More than 50 years later, Gerber et al. noted that adults with irreparable posterosuperior rotator cuff tears have similar abduction and external rotation deficits as children diagnosed with plexus palsy [2]. Therefore, they treated these patients with an isolated LD transfer using the two-incision technique and found good functional results [3, 4]. Boileau et al. modified the L’Episcopo tendon transfer technique by transferring LD combined with TM using the deltopectoral approach [5]. In addition, an isolated TM transfer has been described [6], and more recently, the ventral LD transfer for reconstruction of irreparable anterosuperior rotator cuff tears has been proposed [7–9].

For all tendon transfers around the shoulder, an anatomical understanding of the tendon insertions and relationship between each other is of tremendous importance. In LD tendon transfers, it is required that the LD is released from its insertion and surrounding soft tissue and finally mobilized to minimize tension and potential damage to the adjacent nerves. Any abnormality or interconnection between the transferred tendon and the surrounding tissue may impair tendon mobility. Several authors have therefore studied the relationship between LD and TM, particularly the insertion variants of the tendons [7, 10–18], and described different anatomic variants of TM [19–24]. As such, an additional strand of muscle fibers proximal to TM was observed and named as the TM accessorius [16].

However, to our knowledge, no study has described a distal slip of the TM, which was incidentally observed by the reporting authors during LD transfer surgery. Therefore, the purpose of this study was to investigate the anatomy of the distal teres major slip (dTMs) and to describe its relationship with the TM and LD in fresh-frozen cadaver shoulders.

## Methods

The primary goal of this cadaveric study was to analyze the presence and anatomy of the dTMs in relation to the TM and LD. All donors had given their informed consent to use their bodies for scientific purposes and the specimens were purchased through Science Care (Florida, USA). After ethical approval of this study, no specific consent was required for use of these specimens in this study.

### Dissection

As determined on computed tomography, 16 fresh-frozen cadaver shoulder specimens (seven right and nine left shoulders) of 16 hemitorsi had intact glenohumeral joints. Donors included nine men and seven women, all of which were white Americans, with a mean age of 82 ± 7 years at death. All shoulders were thawed for 24 h at room temperature prior to dissection and dissected using the deltopectoral approach by two fellowship-trained shoulder surgeons (LE, PB). After removing the skin, superficial tissue, and pectoralis major muscle, the tendon of the LD could be identified. The LD and TM tendons were carefully separated from the surrounding tissue. After the LD tendon was separated from the underlying TM at its site of humeral insertion, the LD tendon was harvested to obtain a full view of the TM insertion. The TM muscle was then macroscopically checked for the presence of dTMs. When the dTMs was present, the muscle and tendon were dissected in entirety. Finally, the TM with the attached dTMs were harvested for ex situ photo documentation.

### Cadaveric measurements

Measurements were performed with the arm in the neutral (0° abduction) position. Using a tape ruler, the dimensions of the dTMs and LD tendons and muscles were measured. Before the LD tendon was harvested, the LD tendon length and width at the bony insertion were measured in all specimens. Second, the presence of the dTMs was examined, and when present, tendon location relative to the TM and LD, tendon width at insertion and width in continuity, and full tendon length were measured. If the dTMs was adherent to the LD, the distance from the bony insertion to the adherence was documented.

### Statistical analysis

The Shapiro–Wilk test was applied to test the data for normal distribution. Continuous variables are expressed as mean ± standard deviation. The paired t-test was used to compare the widths of the LD and dTMs tendons as well as the dTMs tendon width at the bony insertion with that in continuity. The alpha level was set to 0.05, and all *p*-values were two-tailed.

## Results

**Fig. 1:**
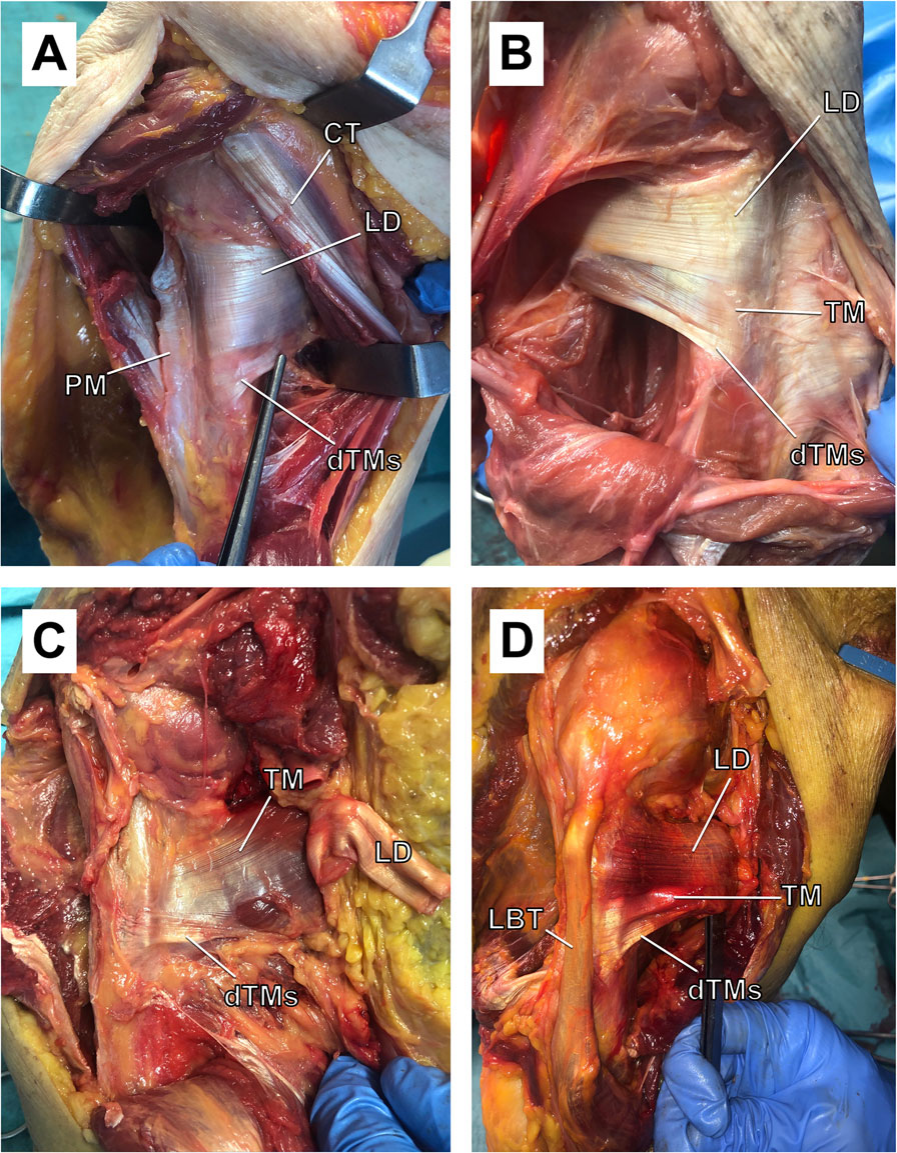
**a** to **d** The anterior view of the humerus after the deltopectoral approach and resection of the pectoralis major tendon. The insertion site of the tendons on the humerus in four different specimens (**a**, **c**, and **d**: left side; **b**: right side) with the presence of the distal teres major slip. Note the separate (distal) but continuous (mediolateral) insertion of the distal teres major slip within a common epimyseal sheet around the TM. LBT, long biceps tendon; LD, latissimus dorsi; PM, pectoralis major; TM, teres major; dTMs, distal teres major slip

In 12 out of 16 (75%) shoulders, the dTMs was present. Its tendon was in 12 out of 12 shoulders distal to the TM tendon and visible during the deltopectoral approach with the LD tendon attached. In the remaining four shoulders (25%), no dTMs was observed, and TM and LD had a common (conjoint) tendon insertion. Once the LD was resected, a clear separation between the TM proximally and the dTMs distally, was observed. The dTMs tendon showed a distinct insertion, which was separate distally from that of the TM insertion and within a common epimyseal sheet around the TM, which formed a continuous humeral insertion (Fig. 1).

Compared to the LD tendon, the width of the dTMs tendon at the bony insertion was half the size (LD, 27 ± 4 mm vs. dTMs, 13 ± 4 mm; *p* < 0.001). The mean dTMs tendon width decreased by more than a third from the bony insertion to the musculotendinous unit (13 ± 4 mm vs. 8 ± 3 mm; *p* < 0.001). The total lengths of the dTMs and LD tendons were 40 ± 7 mm and 69 ± 7 mm, respectively (*p* < 0.001). In 10 of 12 (83%) shoulders, the dTMs muscle showed direct adhesions to the LD muscle. These band-like connections (connectiones intertendineae) were located at a mean distance of 50 ± 8 mm proximal to the bony insertion of dTMs (Table 1).

**Table 1:** Dimensions of the latissimus dorsi and distal teres major slip^a^

	Tendon width at insertion (mm)	Tendon width in continuity (mm)	Total tendon length (mm)	Distance to adhesions with LD (mm)
dTMs (*n* = 12)	13 ± 4 (7–22)	8 ± 3 (4–13)	40 ± 7 (25–57)	50 ± 8 (39–64)^b^
LD (*n* = 16)	27 ± 4 (22–34)		69 ± 7 (48–83)	
*p*-value	0.001		0.001	

*LD* Latissimus dorsi, *dTMs* Distal teres major slip

^a^Data are expressed as mean ± standard deviation and range

^b^*n* = 10; two shoulders had no band-like adhesions with the latissimus dorsi

**Fig. 2:**
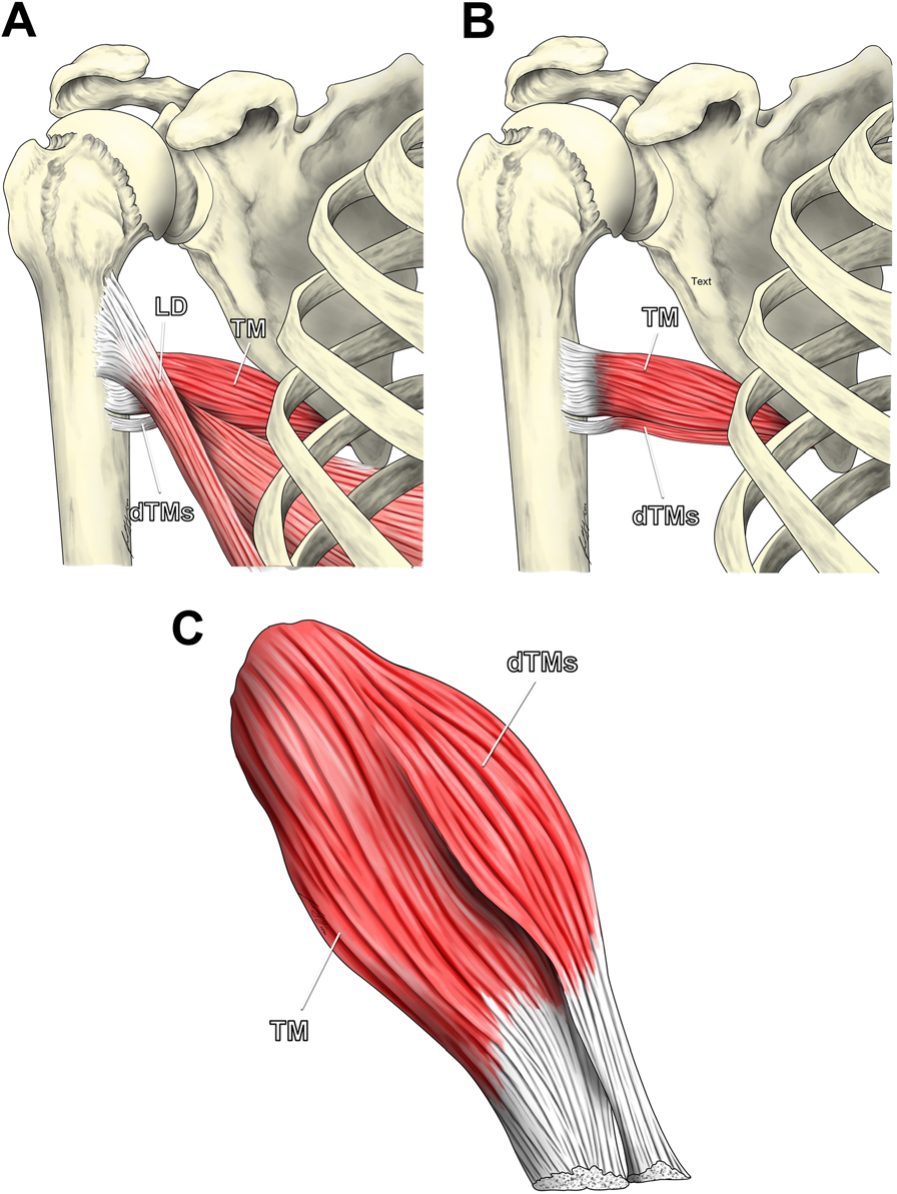
**a** to **c** Schematic drawing of the latissimus dorsi (LD), teres major (TM) and distal teres major slip (dTMs) muscles and tendons. **a**: With the LD tendon attached, the dTMs insertion distally to the LD and TM tendons is visible. **b**: The drawing shows the separate but continuous insertion of the teres major and dTMs tendons after detachment of the LD tendon. **c**: Note the ventral location of the dTMs muscle belly relative to the TM muscle belly

After resection of the TM and dTMs muscles, the muscle belly of the dTMs was ventral to that of the TM muscle and the tendons of both muscles were separate (Fig. 2).

## Discussion

In the present anatomical study, we introduced and described a muscle–tendon slip distal to the TM, which seems to be an additional head of the TM muscle. The dTMs has a separate (distal) but continuous (mediolateral) insertion at the humerus within a common epimyseal sheet around the TM. Other than the previously described TM accessorius [16], which lies proximally, the described additional head is located distal to the TM. This distal teres major slip was identified in 75% of our shoulder specimens, and its presence was defined by a distinct and separate bony insertion distal to the tendon insertion of the TM.

The LD tendon attaches more laterally and proximally compared to the TM tendon with the most inferior portion of the insertion area of the TM usually located inferior to that of the LD tendon [16, 25], leaving parts of the TM bony insertion visible during a deltopectoral approach. Yet, separation of the LD and TM tendon insertions during this approach can be challenging. If present, intraoperative exposure of the dTMs tendon may provide an additional distal lead structure for separating the tendon insertions between the LD and TM when using the deltopectoral approach for tendon transfer surgery. In particular, for isolated LD transfers [5], where the TM is left at its anatomical position, an exact differentiation of the insertion sites between LD and TM is fundamentally important to perform a correct tendon transfer.

Beger et al. classified the interconnections between LD and TM into five different types [17]: type 1 referred to no muscular or tendinous connections, type 2/3 to muscular connections, and type 4/5 to tendinous connections. Wang et al. described additional muscle fibers, which cover the TM tendon posteriorly and attach directly to the periosteum of the humerus [10]. Moreover, Dancker et al. described an additional strand of muscle fibers proximal to TM [16]. The authors assumed an additional head of the TM with considerable thickness and separate bony insertion by a short tendon and named it TM accessorius. Also, several studies reported on additional/anomalic slips of the TM which pass between TM and the long head of the triceps [19–21], or between TM and the fascia of the shoulder capsule and the brachialis fascia [19, 22], or from the biceps brachii to the TM [23], or from the tendon of coracobrachialis to the TM [19], or even from the infraglenoid tubercle to the TM [24]. However, to the best of our knowledge, this is the first study describing an additional distal muscle slip of the TM. In contrast to the TM accessorious, which inserts proximally and medially to the main insertion of the TM [16], the dTMs inserts as a separate and distal tendon with a continuous insertion at the humerus within a common epimyseal sheet around the TM.

Several surgical techniques have been described to transfer LD in cases of posteroinferior rotator cuff insufficiency, with or without TM [2, 5, 26, 27]. More recently, an isolated LD transfer was favored in concomitant reverse total shoulder arthroplasty, particularly in cases of combined pseudoparalysis [28, 29] of active elevation and external rotation [30–32]. This might be even more beneficial in patients with subscapularis muscle insufficiency, to prevent further weakening of the internal rotation force of the shoulder with a transformation of two internal rotators into external rotators. The LD tendon transfer can be performed using a single [5, 26, 30–34] or double incision technique [5, 26, 30–34]. In particular, for the single incision technique, the findings of the present study can contribute to improving the surgical safety by providing a deeper understanding of the surgical anatomy. We identified a separate muscle belly of the TM with a separated and more distal tendon insertion in three-quarters of the shoulder specimen examined. A specific band-like connection between the LD tendon and dTMs was found to be located approximately 5 cm proximal to the tendon insertion. A fascial-like connection between LD and dTMs has been described previously [14]. However, we could describe the distinct location of this important connection that needs to be released (together with the interconnections between LD and TM) in isolated LD tendon transfers in order to achieve appropriate tendon excursion and to minimize tension. Moreover, it remains controversial whether an isolated single-incision LD transfer should be performed underneath TM [31] or through a split within the muscle, as introduced by Popescu et al. [30]. The LD transfer passage within the TM muscle seems to be feasible just between the TM and dTMs. Comparing the figures of that study to our findings, it remains possible that the transfer was actually performed in the study by Popescu et al. [30]. Yet, it remains necessary to study how well and to what extent the TM and dTMs can be separated from each other without jeopardizing them.

In patients with irreparable subscapularis tears, pectoralis major transfer [35–37] has been the most commonly performed procedure for glenohumeral joint preservation with good to excellent long-term results [38]. More recently, ventral LD transfer has been described as an alternative technique for irreparable subscapularis tears [7, 9]. As in the single-incision LD transfer for posterosuperior tears, locating and harvesting the tendon in a ventral LD transfer are performed similarly. The findings of this anatomic study could help to improve the safety and efficiency of ventral LD transfers in patients with irreparable subscapularis insufficiency.

A limitation is that we could not study the function as well as neurovascular supply pattern of the dTMs, as it was not feasible in our cadaveric shoulder specimen. The lack of tone and neuromuscular control and the alteration of elasticity due to the freeze-thaw process should also be noticed when interpreting our results. The macroscopic appearance and excursion are suggestive of an additional distal teres major muscle slip. However, in order to confirm the dTMs, further anatomical studies (of function, innervation patterns, histology, and pennation angles) are necessary. Further, we did not evaluate whether the dTMs was present unilaterally or bilaterally, as only one shoulder specimen of each donor was available. We also did not study the presence of the dTMs in different ethnic groups than white American. Therefore, the presence of the dTMs may be the result of ethnical specificity and needs to be further investigated. The authors believe that this new anatomic knowledge is of importance in the field of tendon transfers around the shoulder.

## Conclusions

This is the first macroscopic description of an additional distal slip of the teres major muscle. The dTMs has a separate (distal) but continuous (mediolateral) insertion at the humerus within a common epimyseal sheet around the TM. The dTMs tendon is visible during the deltopectoral approach and can therefore provide a lead structure, particularly in ventral LD transfers with the deltopectoral approach.

## Data Availability

Not applicable.

## Abbreviations

TM: Teres major
dTMs: Distal teres major slip
LD: Latissimus dorsi
